# Theoretical study on the prediction of optical properties and thermal stability of fullerene nanoribbons

**DOI:** 10.1038/s41598-024-80338-w

**Published:** 2024-11-22

**Authors:** Haonan Bai, Xinwen Gai, Lulu Sun, Ji Ma

**Affiliations:** College of Science, Liaoning Petrochemical University, Fushun, 113001 People’s Republic of China

**Keywords:** Nanophotonics and plasmonics, Optics and photonics

## Abstract

In this work, we predicted two different configurations of fullerene nanoribbons (quasi-hexagonal phase (qHP) and quasi-tetragonal phase (qTP)) based on two-dimensional fullerenes, with widths of 1, 2, and 3 fullerene units, respectively. Based on first-principles calculations and ab-initio molecular dynamics (AIMD), the thermal stability and optical properties of six fullerene nanoribbons were predicted. AIMD studies indicate that wider qHP nanoribbons (qHPs) exhibit better thermal stability, while increased temperatures lead to greater instability. In contrast, qHP-3 shows the best thermal stability among the six structures. Then, the optical gap between the calculated and experimental quasi-hexagonal two-dimensional fullerenes is compared to illustrate the accuracy of the calculation. The absorption spectra of six fullerene nanoribbons were calculated and the anisotropy of light absorption was analyzed. Finally, the charge transfer modes of each excited state were visualized through electron-hole density plots. This work provides an essential theoretical foundation for understanding new all-carbon materials, specifically fullerenes.

**Supplementary Information:**

The online version contains supplementary material available at 10.1038/s41598-024-80338-w.

## Introduction

Carbon exists widely in nature, and they exist in different forms of simple substances and compounds. Carbon materials have extremely strong heat resistance, and their softening temperature is generally between 800 and 1200 °C. Its low heat transfer coefficient enables carbon materials to be used normally at higher temperatures, and the mechanical properties of carbon materials can also be adjusted through heat treatment. The stability of the materials is related to temperature, and the higher the temperature, the greater the vibration amplitude. Therefore, the higher the temperature, the more unstable the carbon material. The thermal stability of carbon materials is positively correlated with the firmness of C-C chemical bonds, and the firmness of chemical bonds is positively correlated with bond energy. Carbon atoms form different carbon-based nanomaterials through different hybridization methods, that is, various carbon allotropes. For example, the thermal stability temperature of SP^3^ hybridized diamond is 1500 °C, and it is completely transformed into graphite at 2100 °C^1^; the thermal stability temperature of two-dimensional graphyne formed by SP hybridization and SP^2^ hybridization is theoretically predicted to be as high as 1000 °C, and when the temperature reaches 2000 °C, it will turn into graphene^2^; while the thermal stability temperature of carbon nanotubes formed by the hybridization of SP^2^ and SP^3^ is as high as 3000 °C, and the thermal stability temperature of the same hybrid graphene is about 1000 °C, and the thermal stability coefficient between 0.0200 and 0.0013^3,4^. The carbon in the recently synthesized two-dimensional fullerenes are also a combination of SP^2^ hybridization and SP^3^ hybridization.

In 2022, Zheng Jian et al. prepared an atomic-scale 2D polymer fullerene (C_60_) and synthesized two closely packed quasi-hexagonal and quasi-tetragonal phases single crystals of magnesium embedded polymer C_60_ by reaction under atmospheric pressure by adjusting the ratio of Mg and C_60_^5^. Then, through the interlayer bond breaking strategy, two kinds of large-size single-crystal two-dimensional carbon materials were prepared, namely Monolayer quasi-hexagonal phase fullerene(qHP) and quasi-tetragonal phase fullerene (qTP). The single layer polymer C_60_ is removed from the qHP single crystal, and a small amount of layered polymer C_60_ is removed from the qTP single crystal. The two atomic-scale 2D polymer C_60_ materials have high crystallinity and unique topological structures. Compared to graphene and free C_60_, single-layer polymer C_60_ has a medium band gap of about 1.6 eV. Once reported, qHP and qTP fullerenes have attracted widespread attention^6–10^.

In fact, there are few examples of research on the thermal stability of fullerene and its compound materials, especially the research on the thermal stability of 2D fullerene crystals. In 2023, Elena et al. synthesized single crystals of the layered polymer (Mg_4_C_60_)_∞_ by chemical vapor transport, and then removed the magnesium with dilute acid to obtain electrically neutral, pure carbon-based macroscopic crystals^11^. The authors studied the thermal conductivity of this material and found that it is much higher than that of C_60_ molecules, a result of the formation of in-plane covalent bonds. Also in 2023, Peng investigated the stability and strength of different structures of monolayer fullerenes through first-principles calculations^12^. Studies have shown that pseudo-hexagonal fullerenes have the worst thermodynamic stability at all temperatures. However, the relatively high dynamic and mechanical stability indicates that pseudo-hexagonal fullerenes are stronger than other phases under various strains due to their strong covalent C–C bonds. This is also the reason why only quasi-hexagonal fullerenes have been successfully exfoliated.

It is well known that organic carbon materials have excellent optoelectronic properties due to the special carrier transport characteristics generated by the unique π-electron system in the conjugated carbon network structure, and are widely used in organic light-emitting diodes (OLEDs)^13–15^, dye-sensitized solar cells^16,17^, organic -Inorganic perovskite solar cells^18–20^ and organic transistors^21–23^ and other fields. However, the dimensions of carbon materials have a huge impact on their optoelectronic properties. Along with the structural change from three-dimensional graphite to two-dimensional graphene and then to one-dimensional carbon nanoribbon, its photoelectric properties also change. At present, zero-dimensional, two-dimensional and three-dimensional fullerene structures have been widely studied^24^, but the research on the photoelectric properties of one-dimensional fullerene is blank. Therefore, we designed six types of one-dimensional fullerene nanoribbons (quasi-hexagonal and quasi-tetragonal phases) based on the structures of two-dimensional fullerene crystals. We investigated the thermal stability and optoelectronic properties of these nanoribbons at different temperatures using ab initio molecular dynamics and first-principles calculations.

## Methods

The first-principles and ab-initio molecular dynamics (AIMD) calculations in this study were conducted entirely using the CP2K software^25^. Given the sufficiently large unit cell, the Gamma point was employed for the calculations. After conducting a convergence test for the cutoff energy (see Fig. S1), the cutoff energy was set to 400 eV. Structural optimizations were carried out using the PBE functional^26^ combined with the DZVP-MOLOPT-SR-GTH basis set^27^, along with DFT-D3 dispersion corrections^28^. The length of the unit cell in the x direction is 18.2 Å, with vacuum layers of 10 Å in both the y and z directions. To reduce boundary effects and ensure more accurate simulation results, the unit cell length in the x-direction was expanded to 72.8 Å for the AIMD simulations, with vacuum layers of 50 Å and 30 Å in the y and z directions, respectively. Based on the GFN1-xtb functional^29^, we performed AIMD simulations for six fullerene nanoribbons at 298.15 K with a time step of 1 fs and a total of 10,000 steps to assess their thermal stability at room temperature. To investigate the decomposition temperatures of two fullerene nanoribbons, AIMD simulations were also carried out at 500 K, 1000 K, 1300 K, and 1500 K, with a time step of 1 fs and a total of 6000 steps. The overall motion of the system was removed using the VMD software, and the root mean square deviation (RMSD) curve was plotted. Subsequently, the AIMD trajectory was loaded into VMD^30^, and structures were extracted every 100 frames. A trajectory overlay map was generated, with different colors representing different time points. In order to obtain reasonable UV-vis absorption spectra, we performed TD-DFT calculations for six fullerene nanoribbons under the PBE0 hybrid functional^31^ and DZVP-MOLOPT-SR-GTH basis set, PBE0 improves the accuracy of traditional functionals in excited-state calculations by incorporating a portion of exact exchange (Hartree-Fock exchange) into density functional theory (DFT). The electron-hole densities of each excited state were calculated using the Multiwfn program^32^ and visualized with the VMD software.

The electron-hole density can clearly represent the transfer process of electrons, and describe the regions where electrons increase and decrease as electron and hole, respectively^33–36^. The electron-hole density is defined as:

1 $$\begin{aligned} \rho ^{{hole}} \left( r \right) = & \sum\limits_{{i \to a}} {\left( {\omega _{i}^{a} } \right)} ^{2} \varphi _{i} \varphi _{i} - \sum\limits_{{i \leftarrow a}} {\left( {\omega _{i}^{{\prime a}} } \right)^{2} } \varphi _{i} \varphi _{i} \\ & + \sum\limits_{{i \to a}} {\sum\limits_{{j \ne i \to a}} {\omega _{i}^{a} \omega _{j}^{a} \varphi _{i} \varphi _{j} - } } \sum\limits_{{i \leftarrow a}} {\sum\limits_{{j \ne i \leftarrow a}} {\omega _{i}^{{\prime a}} \omega _{j}^{{\prime a}} \varphi _{i} \varphi _{j} } } \\ \end{aligned}$$

2 $$\begin{aligned} \rho ^{{ele}} \left( r \right) = & \sum\limits_{{i \to a}} {\left( {\omega _{i}^{a} } \right)^{2} } \varphi _{a} \varphi _{a} - \sum\limits_{{i \leftarrow a}} {\left( {\omega _{i}^{{\prime a}} } \right)^{2} } \varphi _{a} \varphi _{a} \\ & + \sum\limits_{{i \to a}} {\sum\limits_{{i \to b \ne a}} {\omega _{i}^{a} \omega _{i}^{b} \varphi _{a} \varphi _{b} - } } \sum\limits_{{i \leftarrow a}} {\sum\limits_{{i \leftarrow b \ne a}} {\omega _{i}^{{\prime a}} \omega _{j}^{{\prime b}} \varphi _{a} \varphi _{b} } } \\ \end{aligned}$$

where $\omega$ is the excitation configuration coefficient, and $\omega^ {\prime}$ is the de-excitation configuration coefficient.* r* is the coordinate vector, $\varphi$ is the orbital wave function,* i* or* j* is the occupied orbital label, and* a* or* b* is the empty orbital label. Thus $\sum\limits_{{i \to a}} {}$ represents every excitation configuration of the cycle, and $\sum\limits_{{i \leftarrow a}} {}$ represents every de-excitation configuration of the cycle. Equation (1) calculates the regions where holes accumulate in the excited state, while Eq. (2) calculates the regions where electrons are formed. These equations visualize the spatial distribution of electrons and holes, enabling a deeper analysis of charge transfer behavior in the excited state.

## Results and discussion

**Fig. 1:**
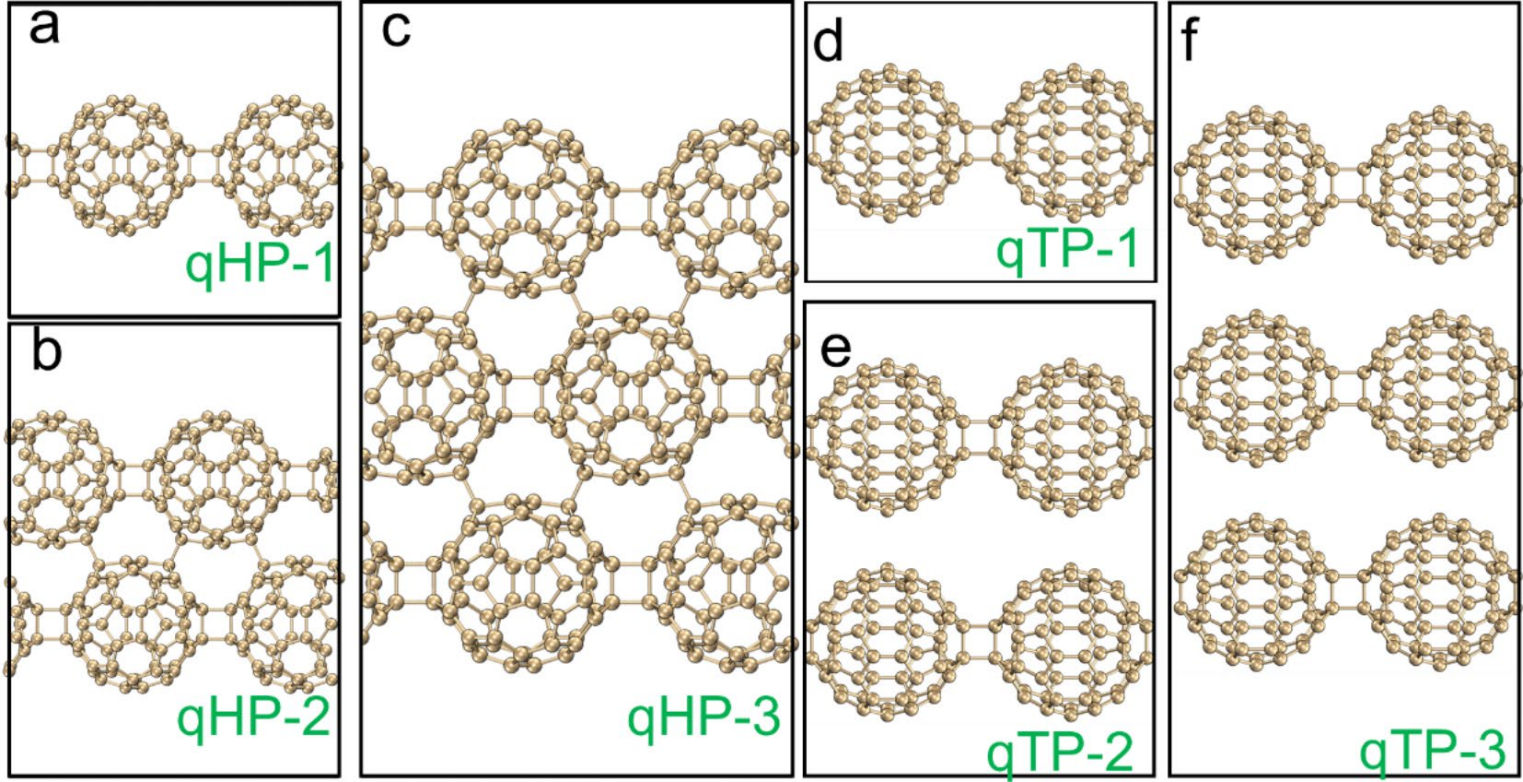
Geometric structures of qHP-1(**a**), qHP-2(**b**), qHP-3(**c**), qTP-1(**d**), qTP-2(**e**) and qTP-3(**f**) .

In this work, existing two-dimensional fullerene crystal structures were initially used as a foundation to create ribbon-like structures of varying widths along the x-axis, containing 1, 2, and 3 fullerene units, respectively. Subsequently, geometric optimization was performed on these nanoribbon structures to ensure their theoretical stability. Subsequently, six fullerene nanoribbon structures with varying widths and symmetries were predicted, as shown in Fig. 1. Among them, Fig. 1a and c depict qHP nanoribbons of different widths, while Fig. 1d and f show qTP nanoribbons with varying widths. Then, six fullerene nanoribbon structures with varying widths and symmetries were predicted. Next, we investigated the thermal stability and optical properties of qHP and qTP nanoribbons. First, the thermal stability of the six structures at temperatures of 300 K, 500 K, 1000 K, 1300 K, and 1500 K was investigated by AIMD. Subsequently, the electronic structures of the highest occupied crystal orbital (HOCO) and the lowest unoccupied crystal orbital (LUCO) were analyzed. Finally, the light absorption properties of fullerene nanoribbons were predicted by periodic TD-DFT, with investigations into the absorption intensity and charge transfer modes of qHPs and qTPs under incident light from various directions.

**Fig. 2:**
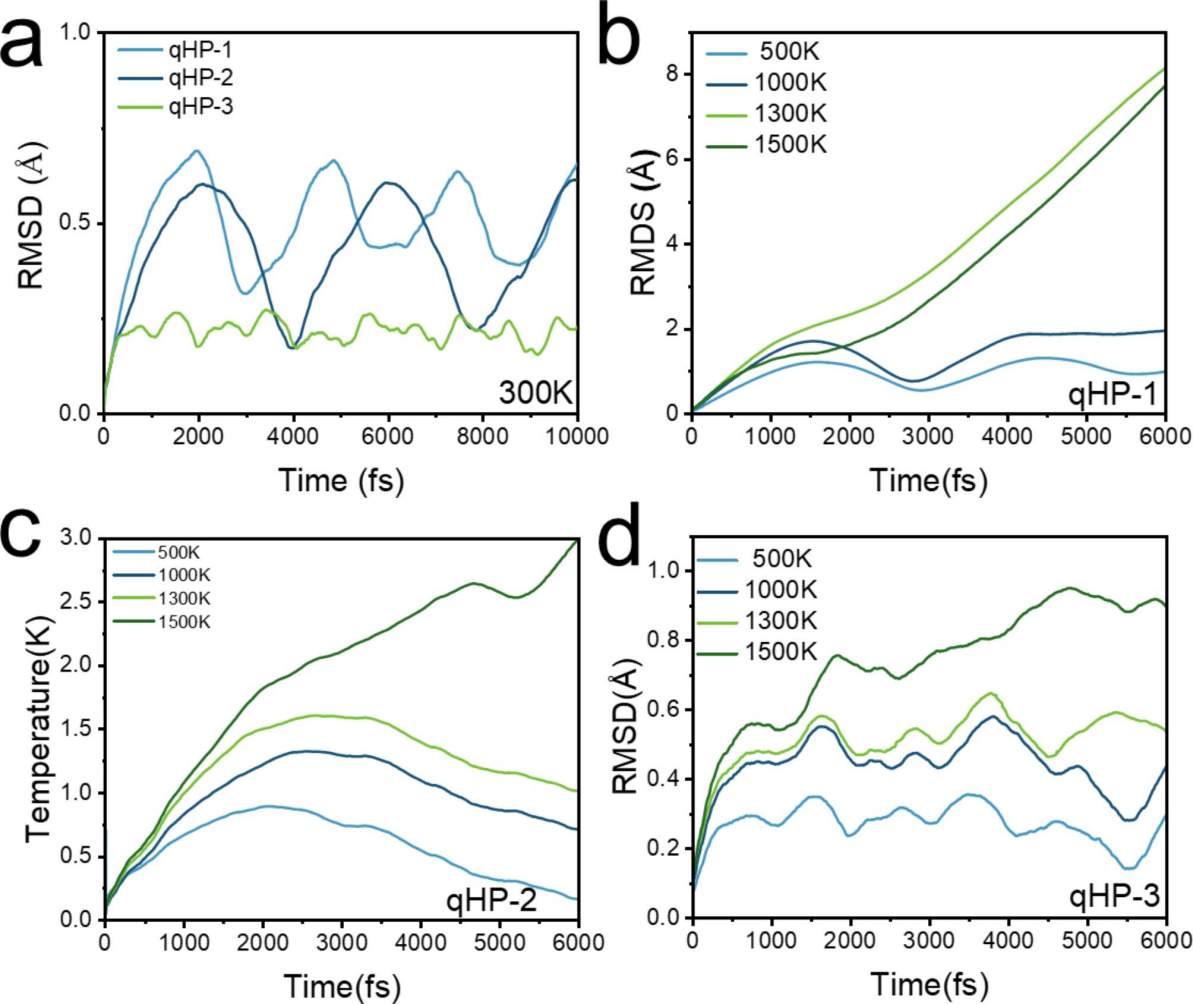
(**a**) RMSD curves of qHP-1, qHP-2 and qHP-3 at 300 K. RMSD curves of qHP-1(**b**), qHP − 2(**c**) and qHP-3(**d**) at 500, 1000, 1300 and 1500 K, respectively.

**Fig. 3:**
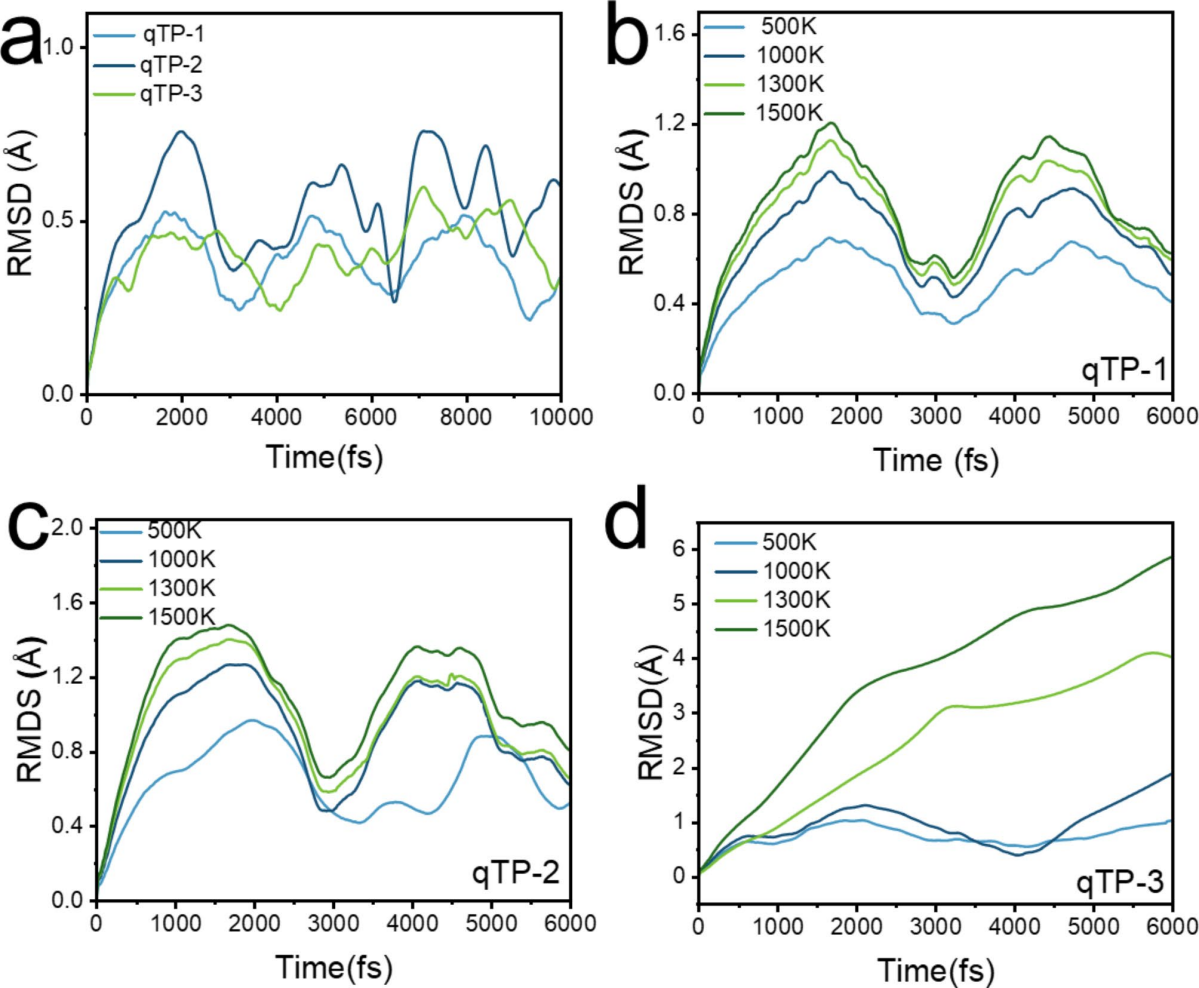
(**a**) RMSD curves of qTP-1, qTP-2 and qTP-3 at 300 K. RMSD curves of qTP-1(**b**), qTP − 2(**c**) and qTP-3(**d**) at 500, 1000, 1300 and 1500 K, respectively.

In order to understand the thermal stability of fullerene nanoribons and the temperature range of cracking into fullerene monomers. First, 10,000 fs AIMD simulations were carried out for qTPs and qHPs at room temperature, and the RMSDs are shown in Figs. 2a and 3a. It can be seen that the RMSD of these six fullerene nanoribbons fluctuates periodically, and the wider the qHP, the smaller the fluctuation range. Among qTPs, qTP-1 is the most stable, and qTP-3 is the most unstable. At the temperature of 300k, the six structures do not break bonds and detach, and the geometric fluctuations are very small. This indicates that all six nanoribbons can exist stably at room temperature^37^. Due to the accelerated atomic motion in the system under high-temperature conditions, thermal fluctuations and potential structural changes occur more rapidly. Therefore, even with fewer simulation steps, the major trends in thermal stability and potential structural changes can still be effectively captured. Based on this, qHPs and qTPs were subjected to AIMD simulations for 6000 fs at 500 K, 1000 K, 1300 K, and 1500 K. This approach allows for an accurate reflection of the thermal motion of fullerene nanoribbons at different temperatures and enables the determination of their cracking temperature. It can be seen from Fig. S2 that during the simulation, the system heats up rapidly within 1000 fs, and the average temperature is very close to the reference temperature. The overall temperature fluctuation is within a reasonable range, indicating that the temperature is maintained well during AIMD simulation. The root-mean-square deviation (RMSD) curves of the six AIMD tracks are shown in Figs. 2 and 3. RMSD trajectories of six fullerene nanoribbons at different temperatures showed three trends.

Under room temperature simulation conditions, the structures of qHPs remain stable, but the stability of qHP-1 is relatively poor. As the width increases, stability gradually improves (see Fig. 2a). At 1300 K and 1500 K, the RMSD trajectory of qHP-1 shows an upward trend, while in the simulations at 500 K and 1000 K, the RMSD curves exhibit periodic fluctuations, with a period of approximately 3000 fs. Therefore, qHP-1 becomes significantly unstable at temperatures above 1300 K (see Fig. 2b). The RMSD trajectory of qHP-2 shows a peak, indicating stronger stability (see Fig. 2c). The repeated rise and fall of RMSD shows that qHP can vibrate periodically and remain stable at this temperature (see Fig. 2d). In contrast, the stability of qHP-3 is significantly enhanced, as the RMSD curve indicates very stable structures. Additionally, the simulation results show that as the simulation temperature increases, geometric fluctuations caused by thermal motion increase significantly. In conclusion, the study demonstrates that the stability of qHPs gradually increases with width. Among the qTP nanoribbons, the RMSD curves of qTPs at 300 K exhibit periodic fluctuations, with qTP-1 and qTP-2 having fluctuation periods of approximately 3000 fs, while qTP-3 has a fluctuation period of around 4000 fs (see Fig. 3a). Therefore, qTP-2 and qTP-3 tend to be more stable. In the temperature range of 500 K to 1500 K, the RMSD curves of qTP-1 and qTP-2 show clear periodic fluctuations with a fluctuation period of approximately 3000 fs, indicating that their stability is superior to qHP-1 and qHP-2 (see Fig. 3b and c). However, at temperatures above 1300 K, the RMSD curve of qTP-3 shows a continuous upward trend, indicating that qTP-3 cannot maintain stability at high temperatures (see Fig. 3d). Thus, the stability of qTP-3 is inferior to that of qHP-3. Additionally, the stability of qTPs decreases with increasing width. The higher the simulation temperature, the greater the geometric fluctuations due to thermal motion. By comparing the RMSD of six C_60_ nanoribbons at 300 K (see Fig. S3), we found that qHP-3 exhibits the best stability.

**Fig. 4:**
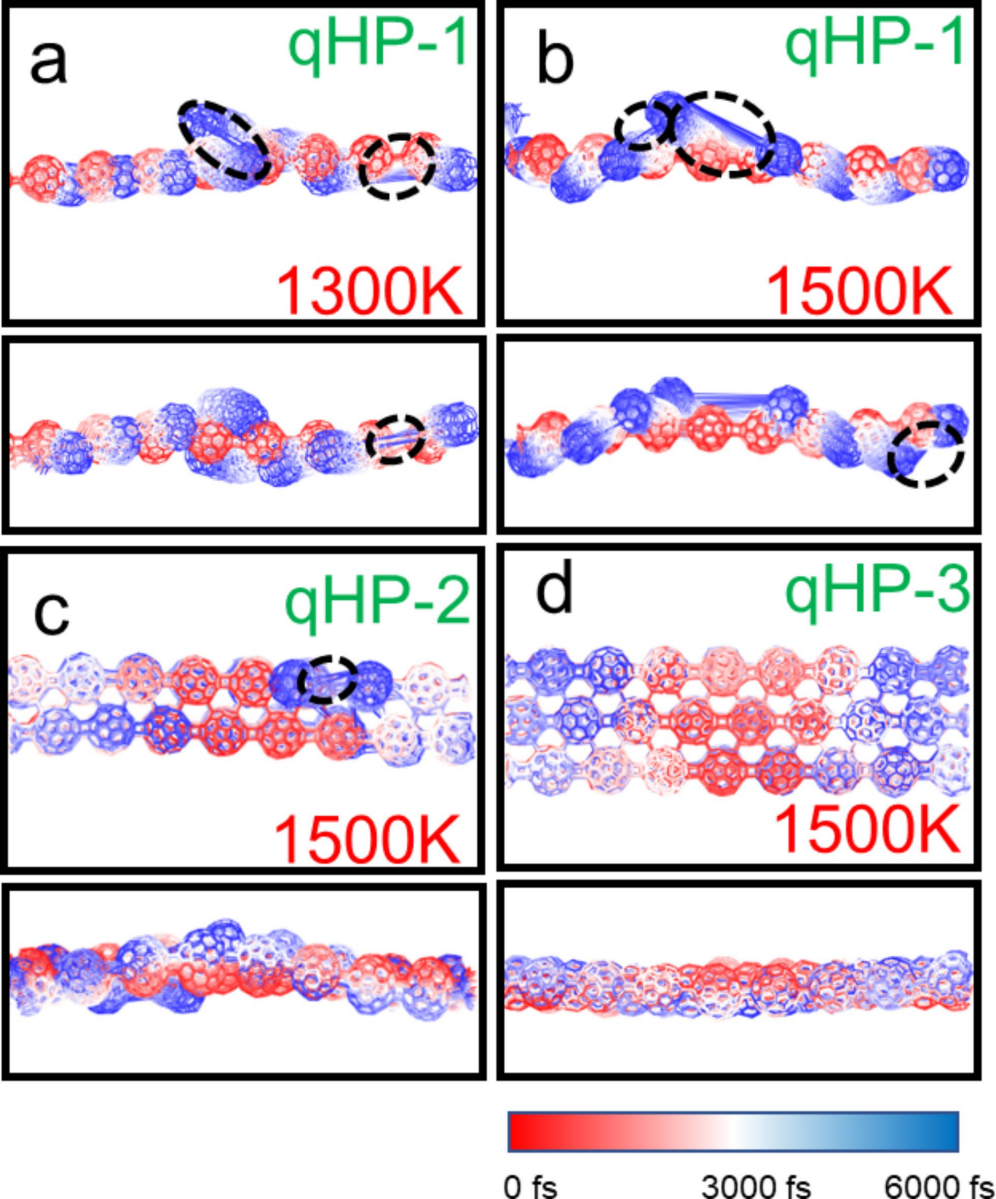
(**a**) AIMD trajectory of qHP-1 at 1300 K, (**b**) AIMD trajectory of qHP − 1 at 1500 K, (**c**) AIMD trajectory of qHP-2 at 1500 K, (**d**) qHP-3 at 1500 K AIMD trajectory. Take a frame structure every 200 frames, a total of 30 frames. Red and blue represent structures in the early and late stages of the simulation, respectively.

We plotted the overlay of the motion trajectories of six fullerene nanoribbons at 500 K, 1000 K, 1300 K and 1500 K. A frame structure is taken every 200 fs from the AIMD trajectory, for a total of 30 frames. The atomic color from red to blue represents the position of the atom in the simulation time from 0 K to 3000 K. It can be observed from the direct grounding in Figs. S3-S9 that the motion amplitude of fullerenes increases with the increase of temperature. qHP-1 undergoes covalent bond cleavage at 1300 K and 1500 K, and qHP-1 decomposes into fullerene monomers and oligomers (Fig. 4a and b). Among them, two covalent bond breaks occurred at 1300 K, and three covalent bond breaks occurred at 1500 K. qHP-2 undergoes one covalent bond break at 1500 K (Fig. 4c). The structure of qHP-3 remained stable at 1500 K, and no large atomic fluctuations occurred (Fig. 4d). The three qHPs at other temperatures also also did not undergo covalent bond cleavage (Fig. S4-S6). It is worth mentioning that the vibration of the three qHP at 500 K is very small, and the structure is very stable. It can be found that the RMSD curve under the three conditions of chemical bond break is constantly rising, which also confirms the previous analysis. The AIMD results show that the three qHP have good thermal stability, and can exist stably at room temperature, even at 500 K. These indicate that qHP can be applied over a certain temperature range.

**Fig. 5:**
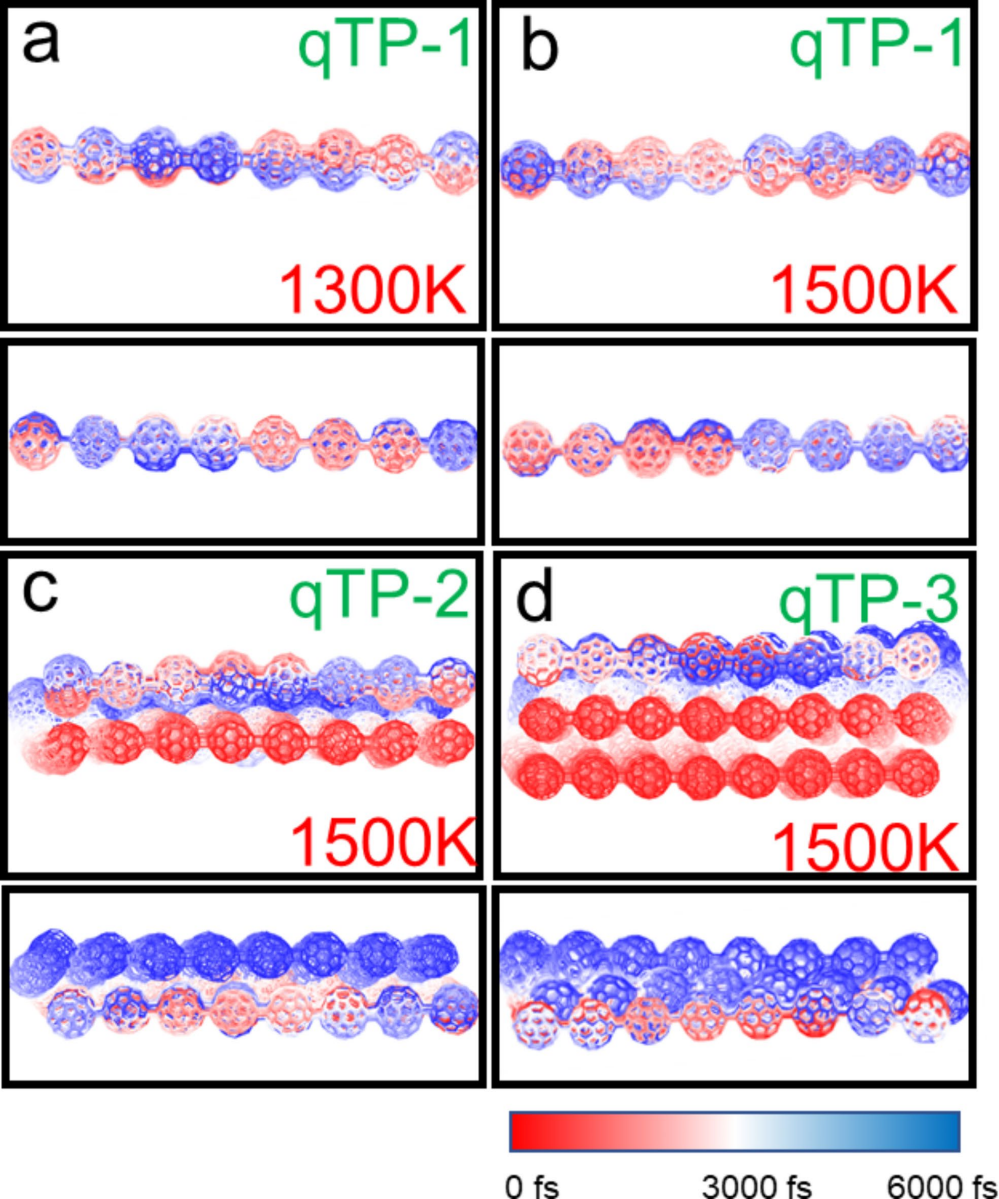
(**a**) AIMD trajectory of qTP-1 at 1300 K, (**b**) AIMD trajectory of qTP − 1 at 1500 K, (**c**) AIMD trajectory of qTP-2 at 1500 K, (**d**) qTP-3 at 1500 K AIMD trajectory. Take a frame structure every 200 frames, a total of 30 frames. Red and blue represent structures in the early and late stages of the simulation, respectively.

The AIMD trajectory of qTPs is shown in Fig. 5 and Figs. S7-S9. It can be observed that no chemical bond cleavage occurs in qHPs at high temperatures, and the fluctuation range of qTP-1 at 1300 K and 1500 K is very small, indicating strong stability (Fig. 5a and b). However, for qTP-2 and qTP-3 at 1500 K, the vibration between the two layers significantly intensifies. Two or three initially aligned fullerenes become noticeably misaligned over time, with the misalignment worsening as the temperature increases (Fig. 5c and d). The vibration amplitude of qTPs at 300 K is also very small, confirming their stable existence at room temperature. However, as the width of qTPs increases, their stability gradually decreases, especially for qTP-3, which cannot maintain stability at temperatures above 1300 K.

**Fig. 6:**
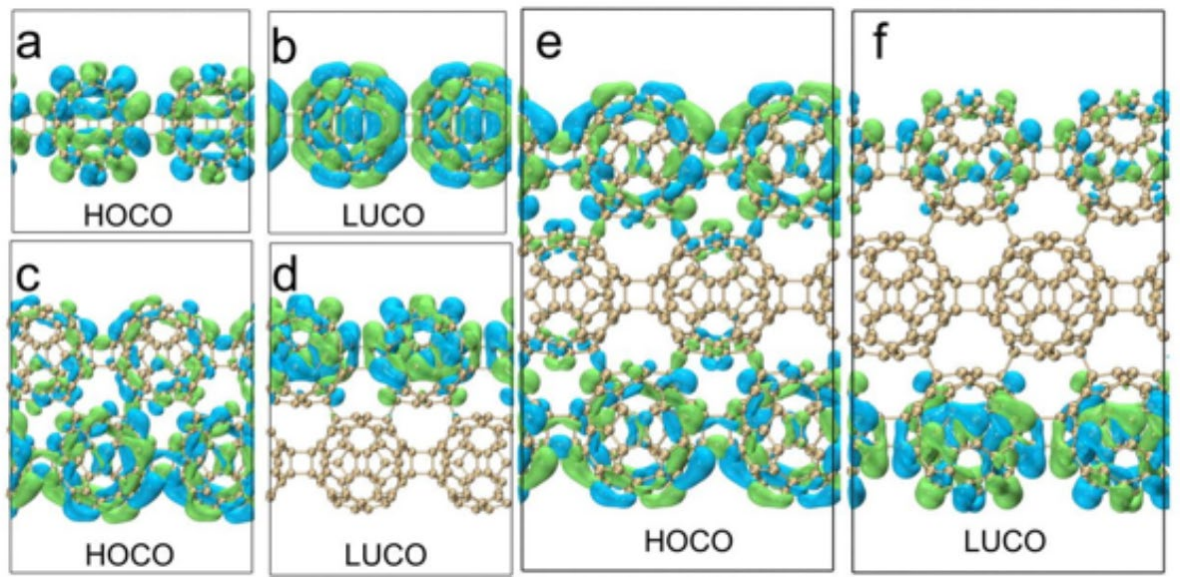
HOCO and LUCO electron distributions of qHP-1(**a**, **b**), qHP-2(**c**, **d**) and qHP-3(**e**, **f**).

Figure 6 shows the isosurface diagrams of the highest occupied crystal orbital (HOCO) and the lowest unoccupied crystal orbital (LUCO) of qHPs. It is calculated that the HOCO-LUCO gap of qHP-1 is 1.32 eV, qHP-2 is 1.11 eV, and qHP-3 is 1.103 eV. It can be observed that with the increase of qHP width, the value of HOCO-LUCO gap is smaller, although this gap is very small. However, it is also proved that qHP is more prone to electronic transition with the increase of width. Both the HOCO and LUCO electron clouds of qHP-1 are distributed throughout the system (Fig. 6a and b). The HOCO and LUCO electron clouds of qHP-2 and qHP-3 are not equally distributed on each fullerene. The HOCO of qHP-2 is mainly distributed in the lower fullerene chain, while a small amount of HOCO is also distributed in the upper part (Fig. 6c and d). The LUCO of qHP-2 is completely distributed on the upper fullerene chain. The HOCO of qHP-3 is evenly distributed on the upper and lower fullerene chains. The LUCO of qHP-3 is mainly distributed in the lower fullerene chain, while the upper fullerene chain has a small distribution (Fig. 6e and f).

**Fig. 7:**
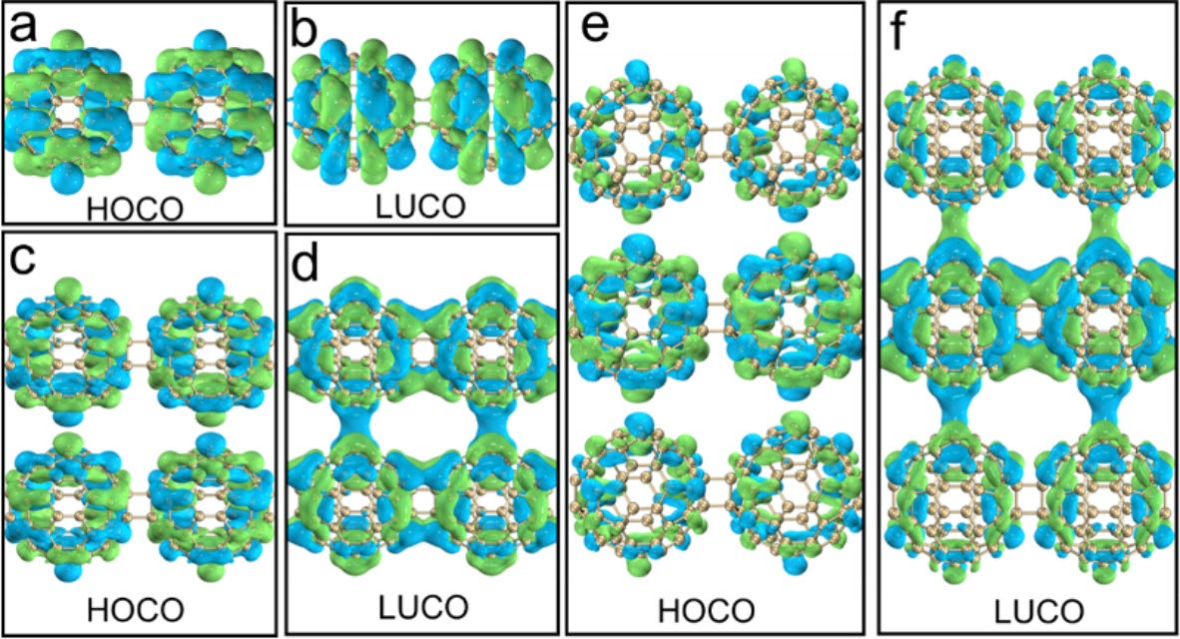
HOCO and LUCO electron distributions of qTP-1(**a**, **b**), qTP-2(**c**, **d**) and qTP-3(**e**, **f**).

Figure 7 shows the isosurface diagrams of HOCO and LUCO of qTPs. The HOCO-LUCO gap of qTP-1, qTP-2 and qTP-3 is 2.26 eV, 2.14 eV, and 2.08 eV, which are consistent with the rule of qHP. The wider the qTP, the easier the electronic transition of qTP is. Comparing qTP with qHP, it is found that the HOCO-LUCO gap value of qHP is smaller, so the electron transition from occupied orbital to empty orbital is stronger in qHP structure. The electron clouds of HOCO and LUCO in qTP-1 are distributed on the fullerene sphere (Fig. 7a and b), the electron clouds of HOCO in qTP-2 are only distributed on the fullerene sphere, and the electron clouds of LUCO are also distributed at the junction of the fullerene sphere, and the electron clouds of HOCO and LUCO are evenly distributed (Fig. 7c and d). The distribution of HOCO and LUCO electron clouds on the fullerenes of qTP-3 is not equal. The fullerenes in the middle layer have more electron clouds, and the fullerenes in the upper and lower layers have less electron clouds (Fig. 7e and f).

**Fig. 8:**
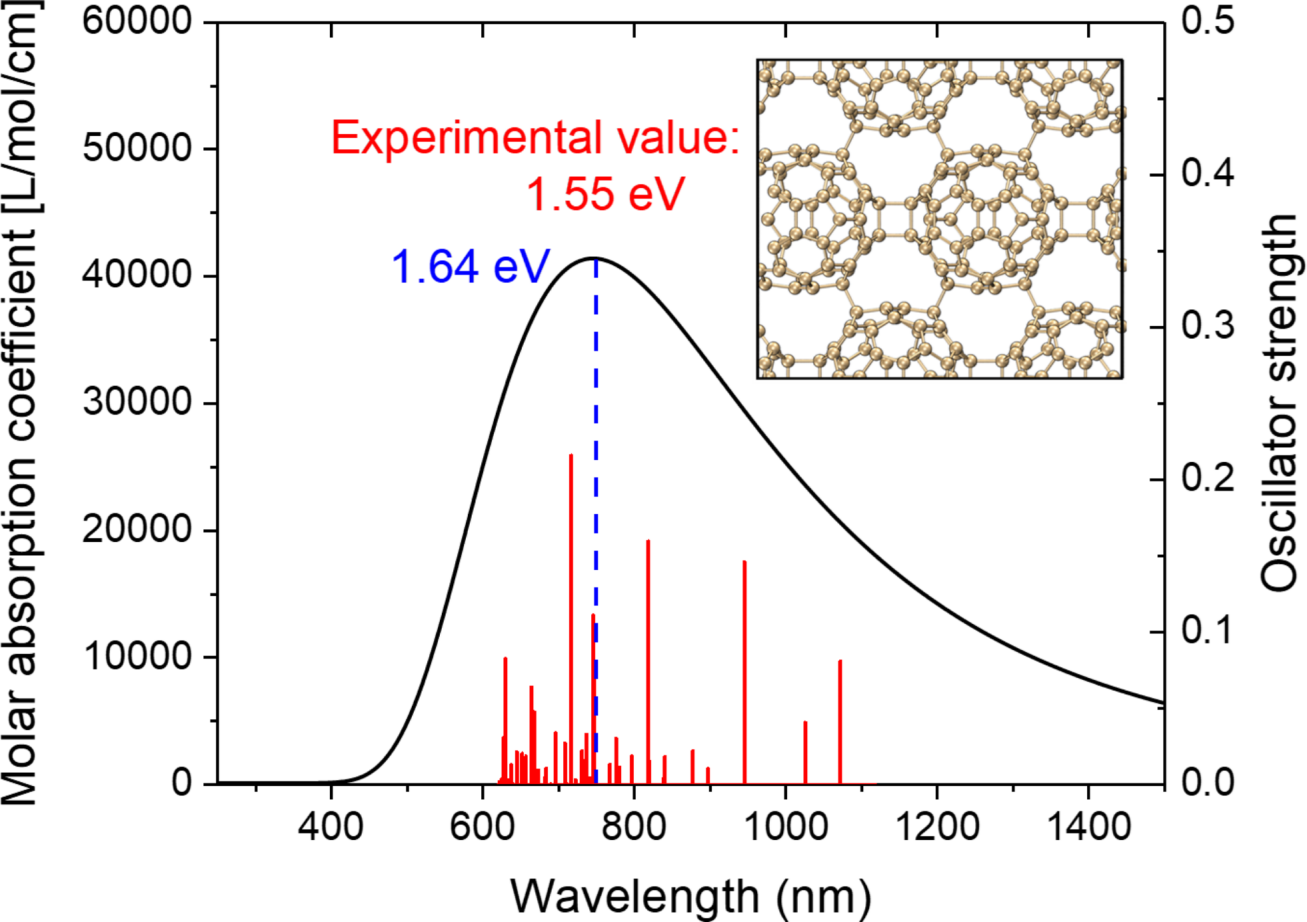
The calculated absorption spectrum of quasi-hexagonal 2D fullerene, the blue text is the calculated optical gap, and the red text is the experimental optical gap.

**Fig. 9:**
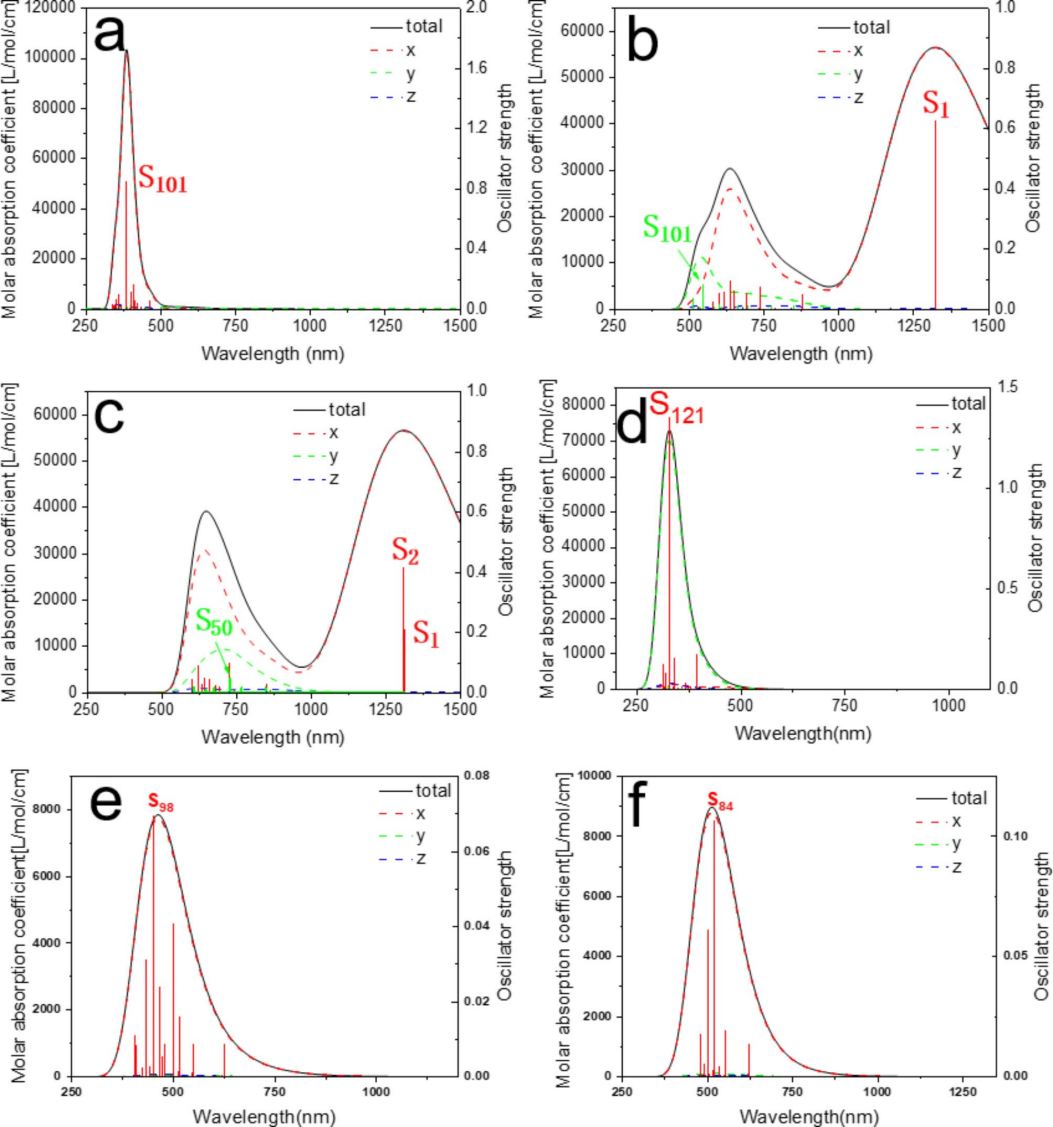
Calculated absorption spectra of qHP-1(**a**), qHP-2(**b**), qHP-3(**c**), qTP-1(**d**), qTP-2(**e**) and qTP-3(**f**). The red, green, and blue curves are components in the x, y, and z directions, respectively.

**Fig. 10:**
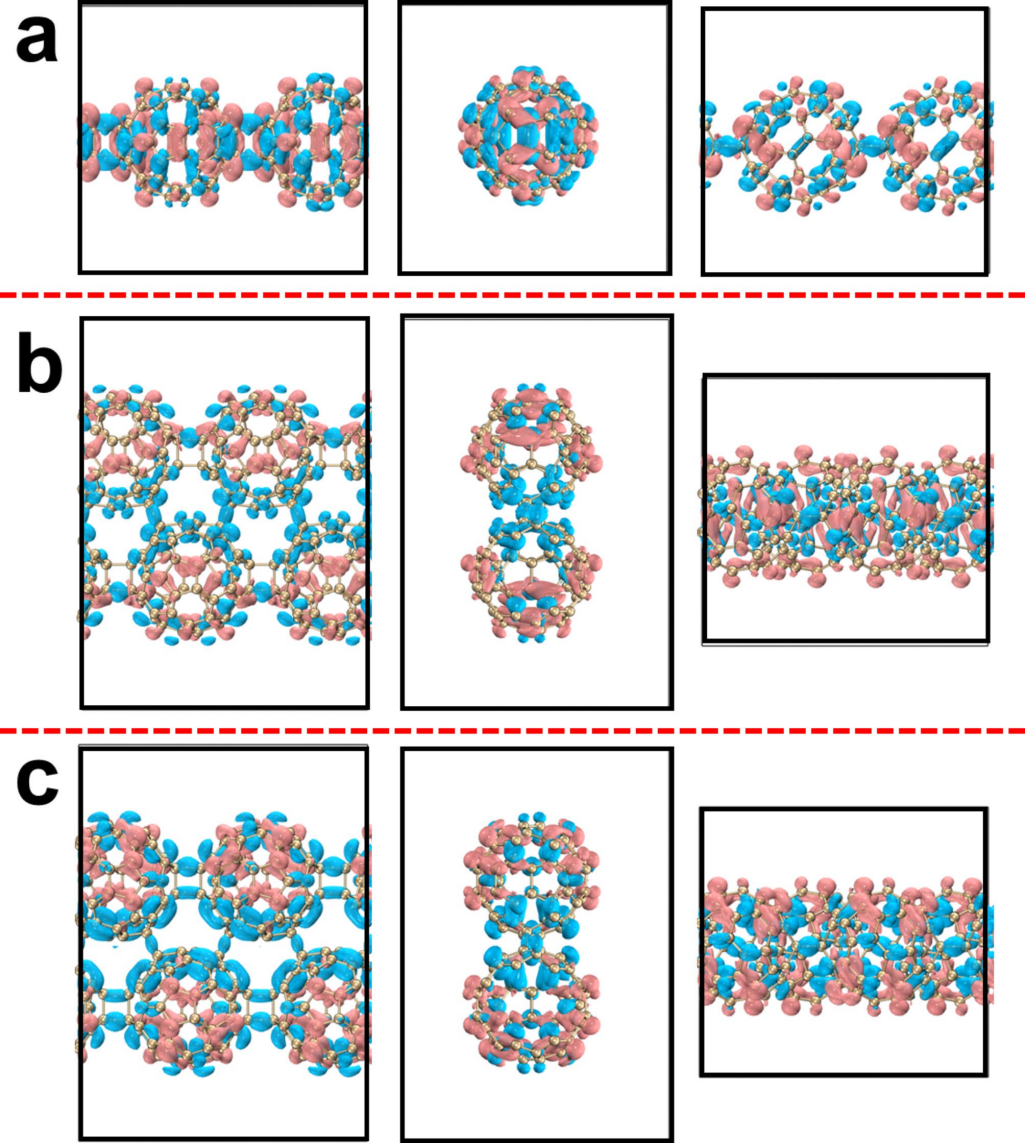
Electron-hole density of qHP-1&S_101_(**a**), qHP-2&S_1_(**b**) and qHP-2&S_101_(**c**), pink and blue represent electrons and holes, respectively.

**Fig. 11:**
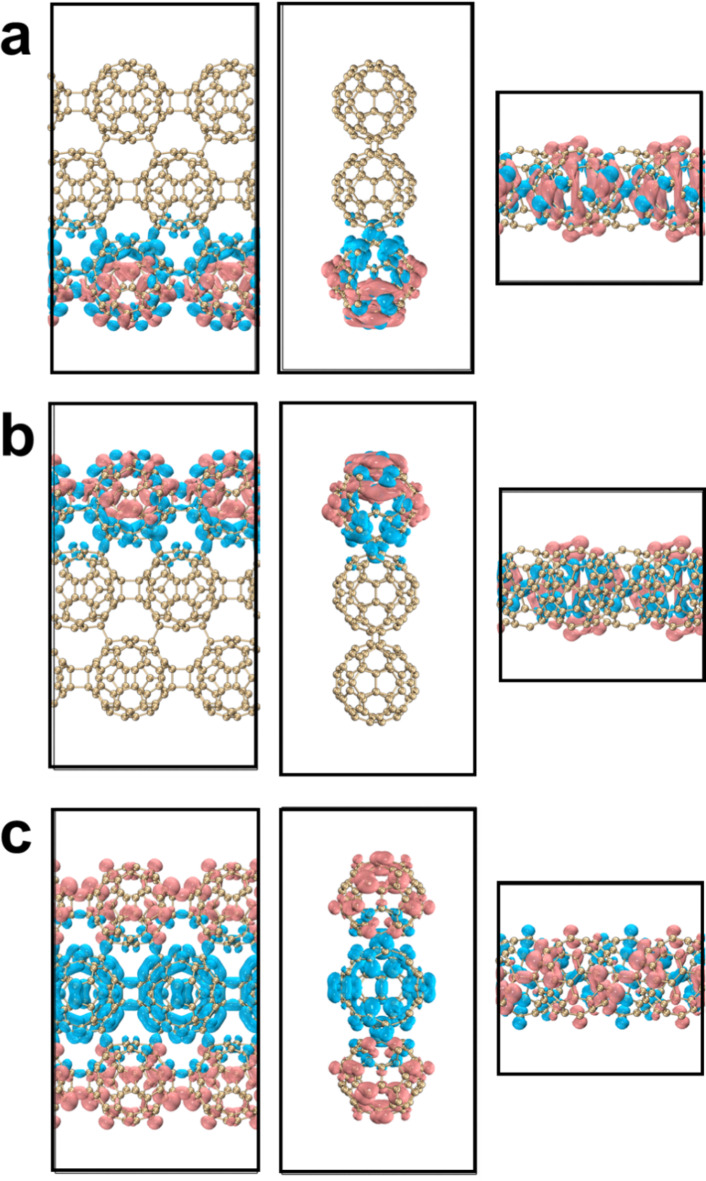
Electron-hole density of qHP-3&S_1_(**a**), qHP-3&S_2_(**b**) and qHP-2&S_50_(**c**), pink and blue represent electrons and holes, respectively.

Next, the light absorption properties of fullerene nanoribbons were studied. Firstly, the absorption spectrum of hexagonal two-dimensional fullerene is calculated at the level of PBE0 functional combined with DZVP-MOLOPT-SR-GTH basis set, as shown in Fig. 8. The optical gap is 1.64 eV. This is only 0.09 eV different from the experimentally measured 1.55 eV, which reflects the rationality of the current calculation level. Figure 9a shows the absorption spectrum of qHP-1 and the upward composnents of each side. It can be seen that there is a strong absorption peak near 370 nm, and this absorption peak is mainly contributed by S_101_. At the same time, the total absorption spectrum is completely coincident with the absorption spectrum in the x direction, indicating that the absorption peak is completely excited by the incident light in the x direction. Figure 10a shows the electron and hole density of the S_101_ excited state of qHP-1, which exhibits strong local excitation. Figure 9b shows the absorption spectrum of qHP-2 and its components in each direction. Different from qHP-1, the absorption spectrum of qHP-2 shows an obvious absorption peak in the Y-direction component. There is a strong absorption peak near 1300 nm, and it is entirely contributed by S_1_, which is caused by incident light in the x direction. Figure 10b shows the electron hole density diagram of the S_1_ excited state of qHP-2. It can be seen that the electron density is mainly distributed on both sides of the fullerene, while the hole density is distributed in the middle position, which indicates that the electrons are transferred from the middle to the two ends. qHP-2 also has an absorption peak near 600 nm, which is caused by incident light in both the x and y directions. The x component is contributed by a large number of small excited states, and the y component is mainly contributed by S_101_. Figure 10c shows the electron hole density of the S_101_ excited state of qHP-2. S_101_ and S_1_ of qHP-2 exhibit the same excitation characteristics, but the charge transfer of S_101_ is significantly greater than that of S_1_. Figure 9c shows the absorption spectrum of qHP-3 and its components in each direction. The absorption spectrum of qHP-3 is very close to that of qHP-2, which has two identical absorption peaks in the same wavelength range. The strong absorption peaks are contributed by S_1_ and S_2_ to the degenerate excited state. Figure 11a and b show the electron hole density maps of S_1_ and S_2_ of qHP-3 respectively. The excitation modes of both are exactly the same, and both are excited by charge transfer from the edge to the inside. The absorption peak of qHP-3 near 600 nm is also contributed by the x component and the y component, and the excited state that contributes mainly to the y component is S_50_. Figure 11c shows the electron hole density diagram of S_50_ of qHP-3, and it can be seen that S_50_ is an obvious excited state of charge transfer. The electrons are distributed on both sides of the fullerene, while the holes are distributed on the fullerene in the middle, indicating that the middle electrons are transferred to both sides.

**Fig. 12:**
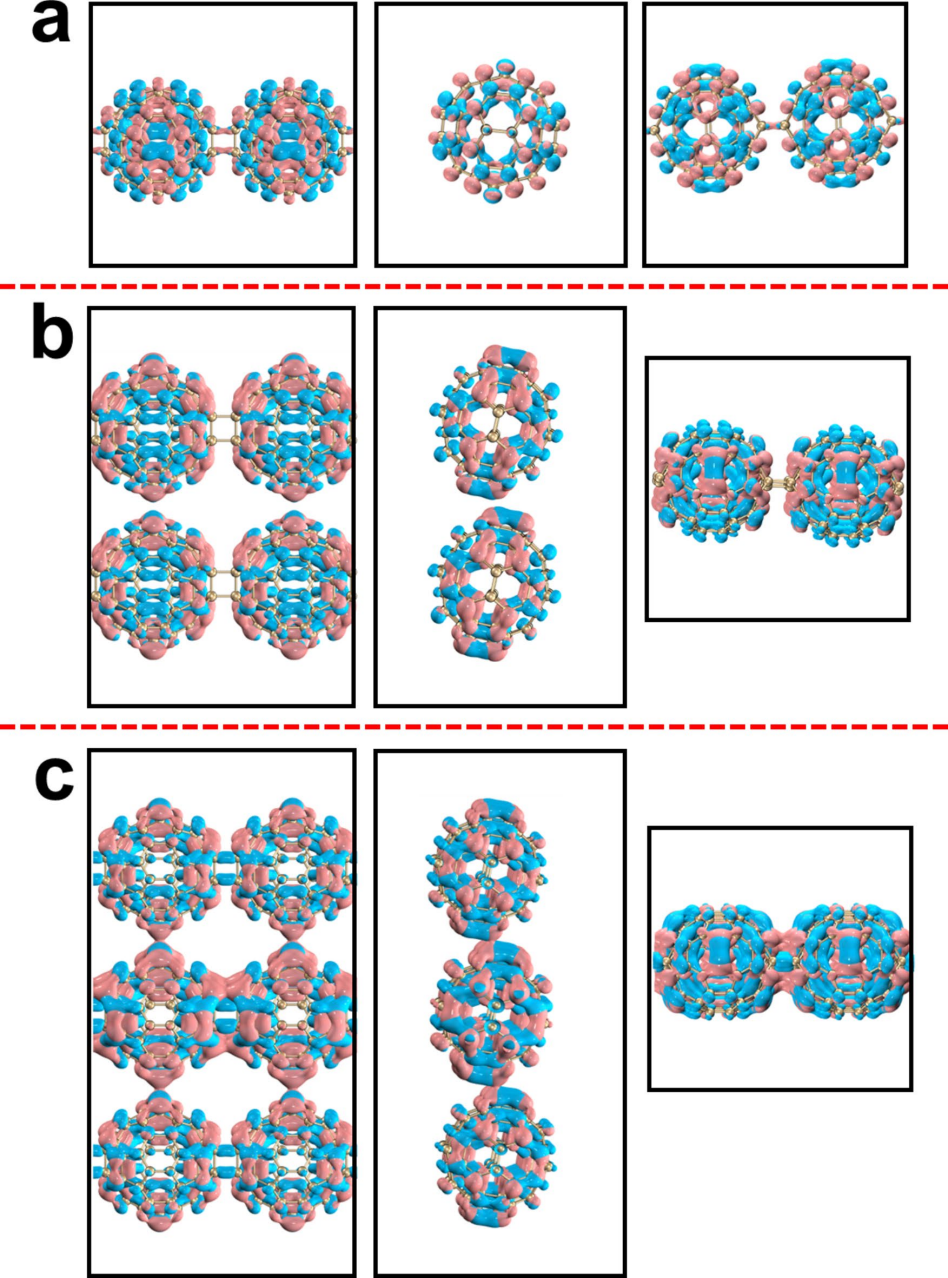
Electron-hole density of qTP-1&S_121_(**a**), qHP-2&S_98_(**b**) and qTP-3&S_84_(**c**), pink and blue represent electrons and holes, respectively.

Figure 9d shows the absorption spectrum of qTP-1 and its components in each direction. Unlike qHP, the absorption spectrum of qTP-1 almost coincides with the spectrum in the y direction, with small contributions from the x and z components. The absorption peak is mainly contributed by S_121_. Figure 12a shows the electron hole density diagram of S_121_ of qTP-1. It can be seen that the holes are on both sides of the fullerene ball and the most central position, and the electrons are between the two layers of holes, which has the property of charge transfer. Figure 9e and f show the absorption spectra of qTP-2 and qTP-3 and their components in each direction, respectively. The absorption spectra of qTP-2 and qTP-3 are very similar. The absorption peak is entirely excited by incident light in the x direction. The absorption peak of qTP-2 is mainly contributed by S_98_. Figure 12b shows the electron hole density diagram of qTP-2, where electrons are distributed around the fullerene and holes are distributed in the center of the fullerene, indicating that electrons are transferred from the center to the periphery. The absorption spectrum of qTP-3 is mainly contributed by S_84_. Figure 12c shows the electron hole density of qTP-3, showing strong local excitation properties.

The light absorption properties of fullerene nanoribbons show strong anisotropy, in which the absorption peaks of qHP-1, qTP-2 and qTP-3 are dominated by the x-direction component. Both qHP-2 and qHP-3 have strong absorption peaks dominated by x-direction components in the infrared range. At the same time, there is an absorption peak near 600 nm contributed by x and y components. Observing the electron hole density map of qHP-2 and qHP-3, it can be found that electrons have a strong ability to transfer outward. However, the absorption peak of qTP-1 is dominated by the y component and contributes little to the x and z directions.

## Results

In this work, we predict the structure of six fullerene nanoribbons and explored their thermal stability and optical properties using first-principles and AIMD methods. First, we simulated the thermal stability of six fullerene nanoribbons at 500 K, 1000 K, 1300 K, and 1500 K using AIMD. The results showed that qHP-3 exhibited the best thermal stability among all the structures. When the temperature increased to 1300 K, qHP-1 underwent cleavage, producing fullerene monomers and oligomers, while qHP-2 cleaved at 1500 K. Additionally, significant misalignment was observed between the layers of qTP-2 and qTP-3. The study demonstrated that the stability of qHPs increases with width, while the stability of qTPs decreases with increasing width. As the temperature rises, the structures of the fullerene nanoribbons gradually become unstable. Then the absorption spectra of the six structures were studied based on TDDFT. Firstly, the absorption spectra of two-dimensional hexagonal fullerenes are calculated, and the optical gap matches the experimental values. This shows the rationality of the calculation. The absorption peak of qHP-1 is located at 380 nm and is completely excited by incident light in the x direction. The absorption spectra of qHP-2 and qHP-3 are very similar, with an absorption peak in the infrared and visible regions, respectively. The absorption peak in the infrared band is excited by the incident light in the x direction, and the absorption peak in the visible region is excited by the incident light in the x direction and the y direction. The absorption spectrum of qTP-1 is contributed by incident light in the y direction. The absorption spectra of qTP-2 and qTP-3 are entirely excited by incident light in the X direction. Then the electron-hole density is used to visualize the charge transfer mode of each excited state. The results show that the electrons in qHP have an obvious inward transfer tendency. While the electrons of qTP-2 are transferred from the inside out, qTP-3 has a strong local excitation property. This work provides the necessary theoretical basis for understanding new all-carbon materials.

## Electronic supplementary material

Below is the link to the electronic supplementary material.

Supplementary Material 1

## Data Availability

Data is available on request from the corresponding author.
