# Supplementary material for: Theoretical study on the prediction of optical properties and thermal stability of fullerene nanoribbons
Source: Sci Rep. 2024 Nov 22;14:28978. doi: 10.1038/s41598-024-80338-w (PMC11584730; doi:10.1038/s41598-024-80338-w)
Supplement: Supplementary file 1 — Supplementary Material 1 [file 41598_2024_80338_MOESM1_ESM.docx]

**Supporting information**


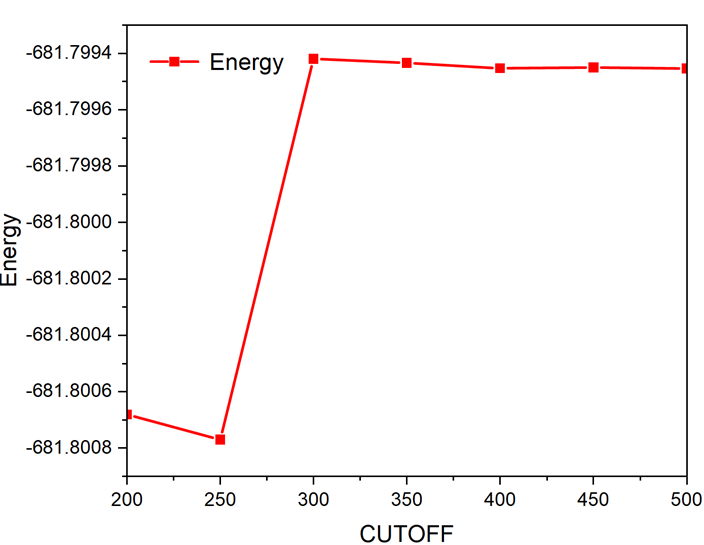


Fig. S1 Convergence test of the cutoff energy.


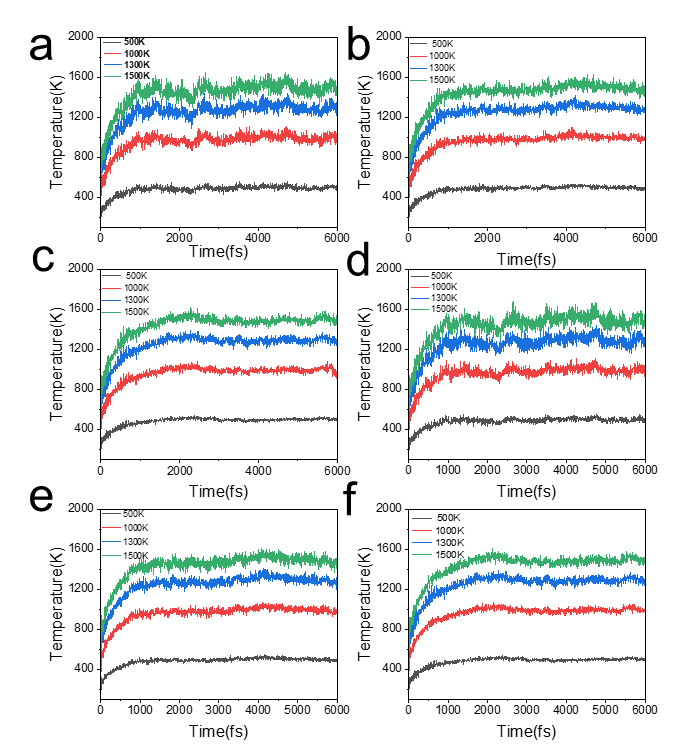


Fig. S2 Temperature curves of qHP-1, qHP-2, qHP-3, qTP-1, qTP-2 and qTP-3, at 500, 1000, 1300 and 1500 K, respectively.


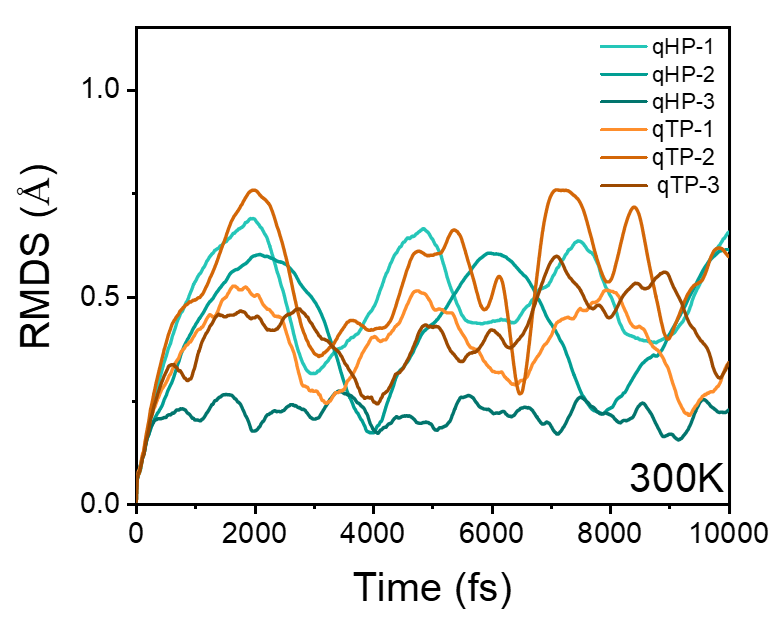


Fig. S3 RMSD curves of qTP-1, qTP-2, qTP-3, qHP-1, qHP-2 and qHP-3 at 300K.


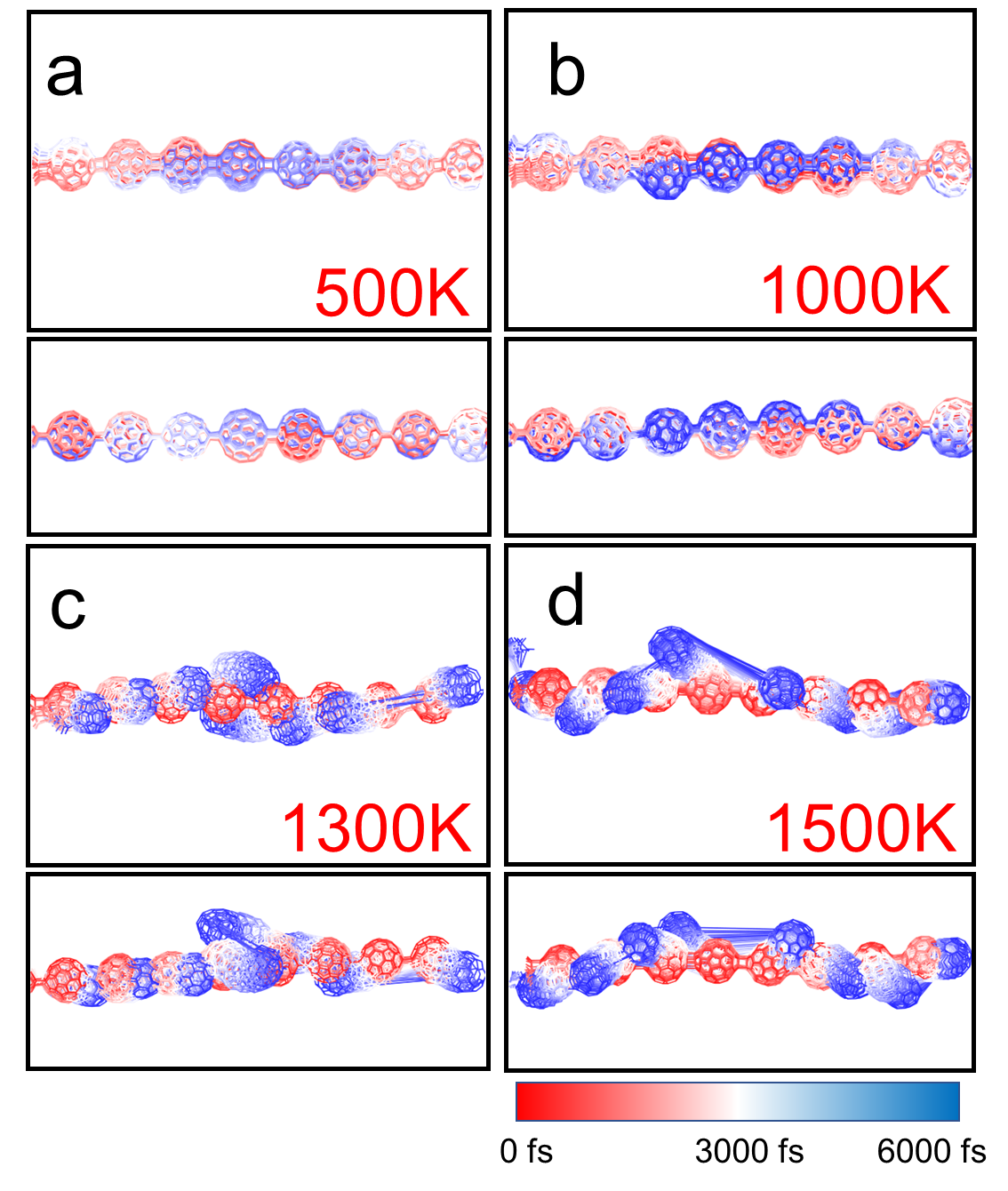


Fig. S4 AIMD trajectories of qHP-1 at 500, 1000, 1300 and 1500K, respectively. Red and blue represent structures in the early and late stages of the simulation, respectively.


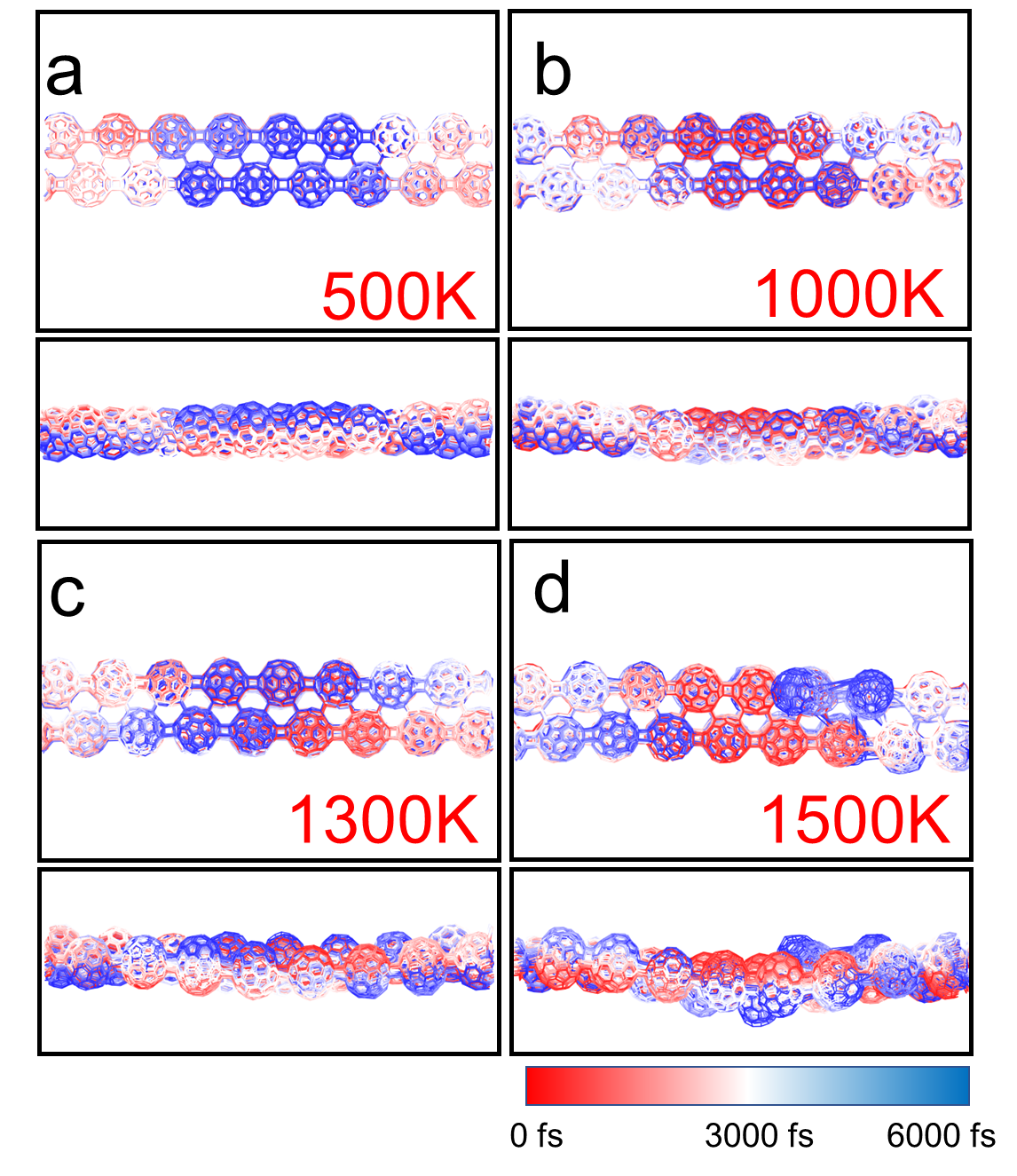


Fig. S5 AIMD trajectories of qHP-2 at 500, 1000, 1300 and 1500K, respectively. Red and blue represent structures in the early and late stages of the simulation, respectively.


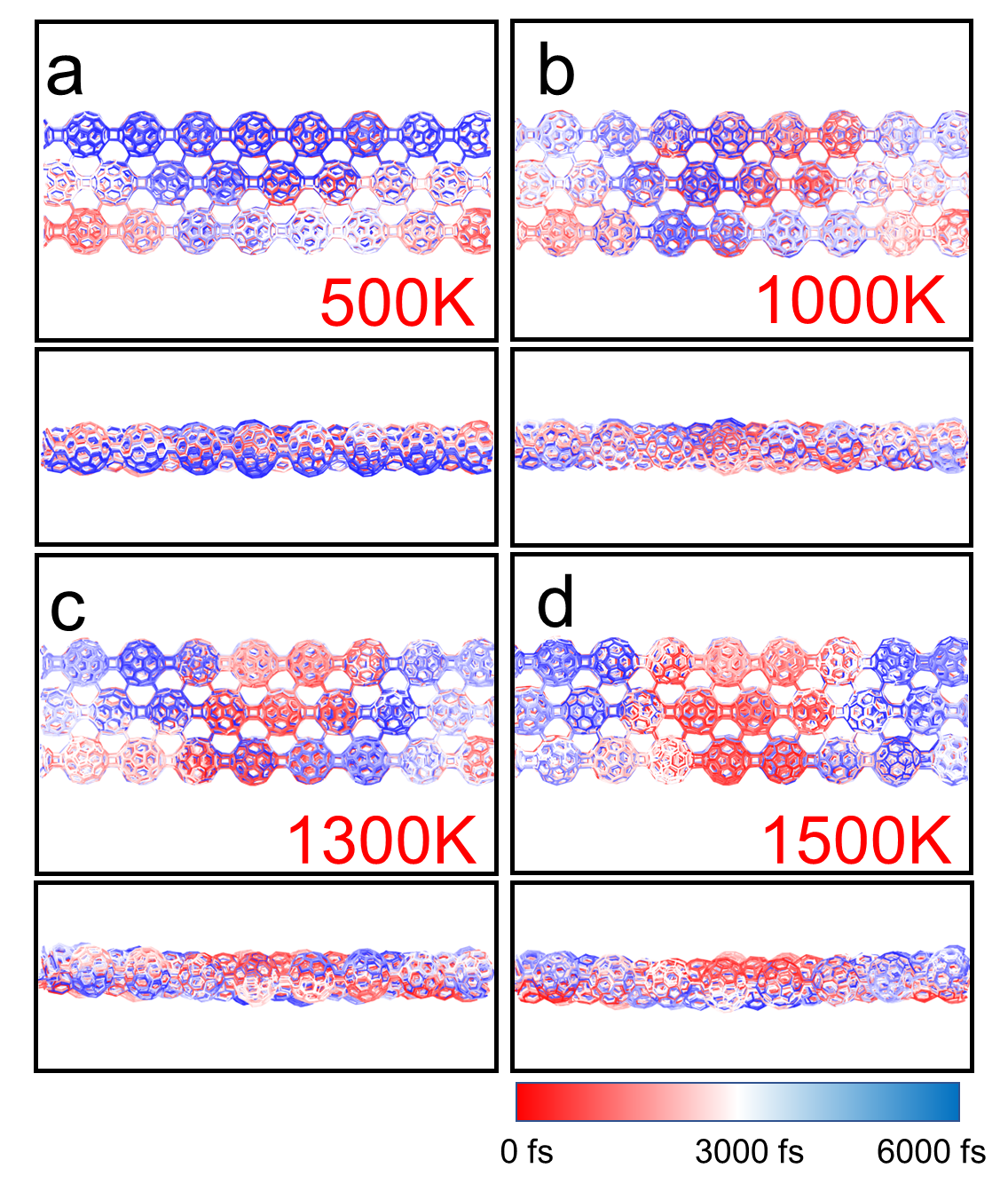


Fig. S6 AIMD trajectories of qHP-3 at 500, 1000, 1300 and 1500K, respectively. Red and blue represent structures in the early and late stages of the simulation, respectively.


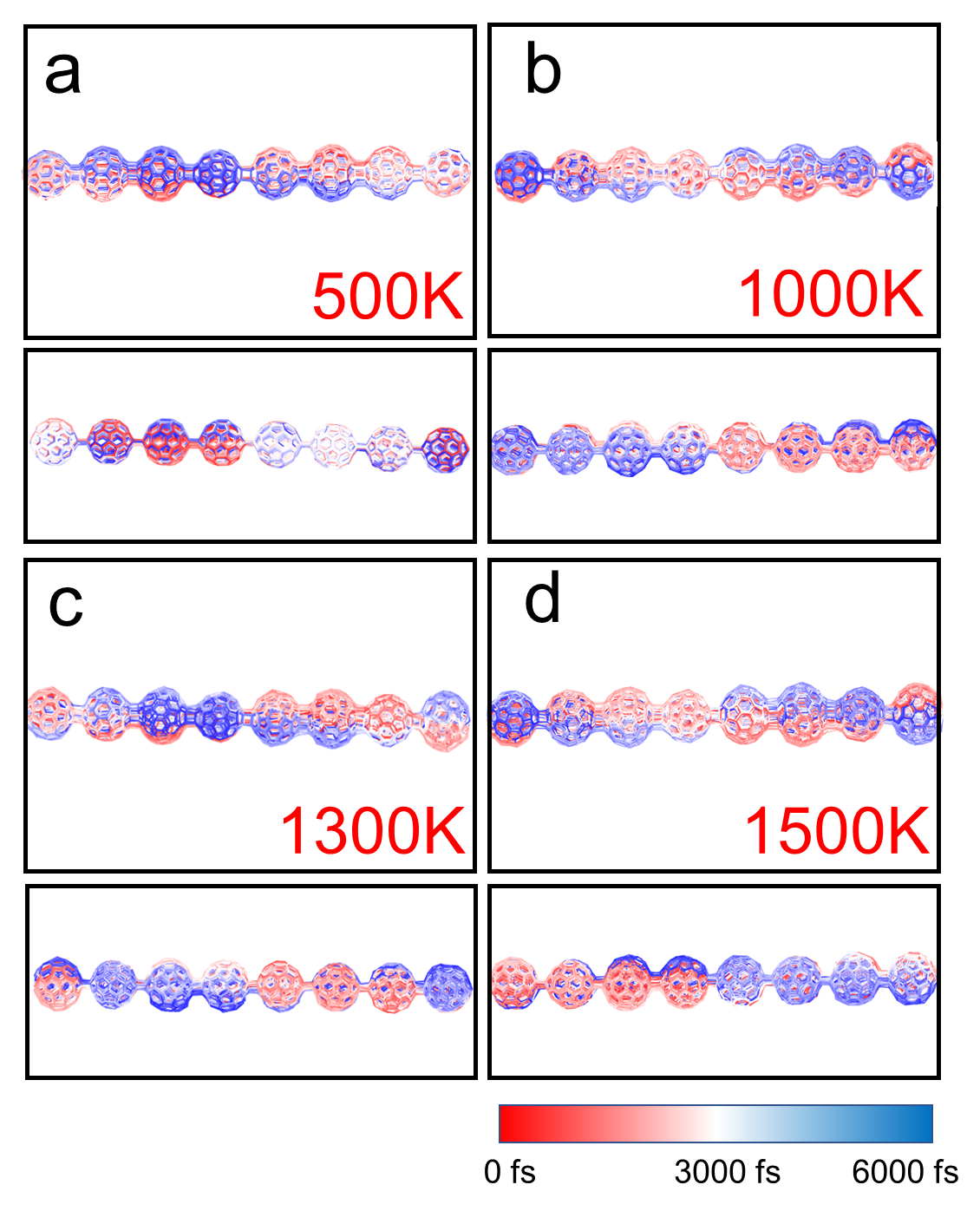


Fig. S7 AIMD trajectories of qTP-1 at 500, 1000, 1300 and 1500K, respectively. Red and blue represent structures in the early and late stages of the simulation, respectively.


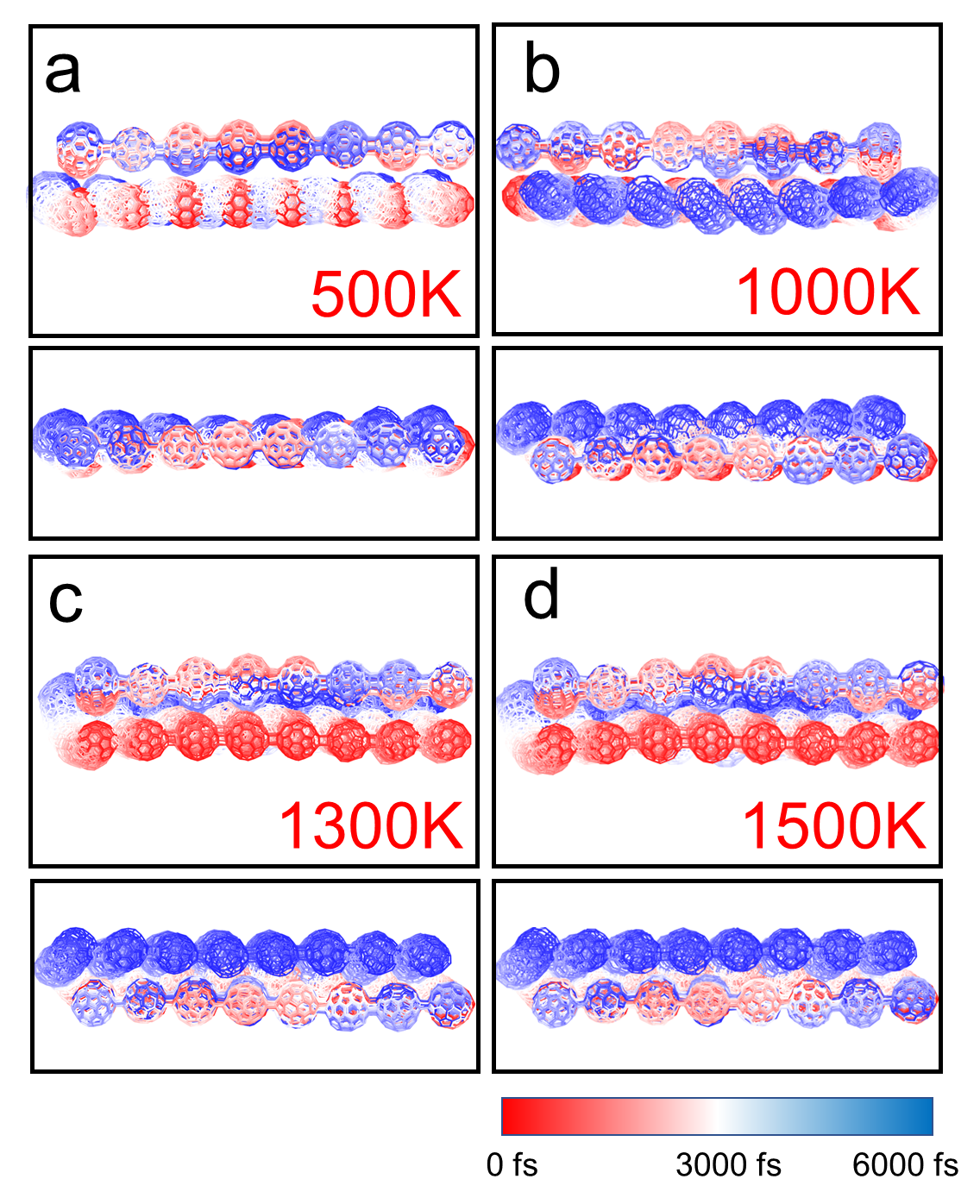


Fig. S8 AIMD trajectories of qTP-2 at 500, 1000, 1300 and 1500K, respectively. Red and blue represent structures in the early and late stages of the simulation, respectively.


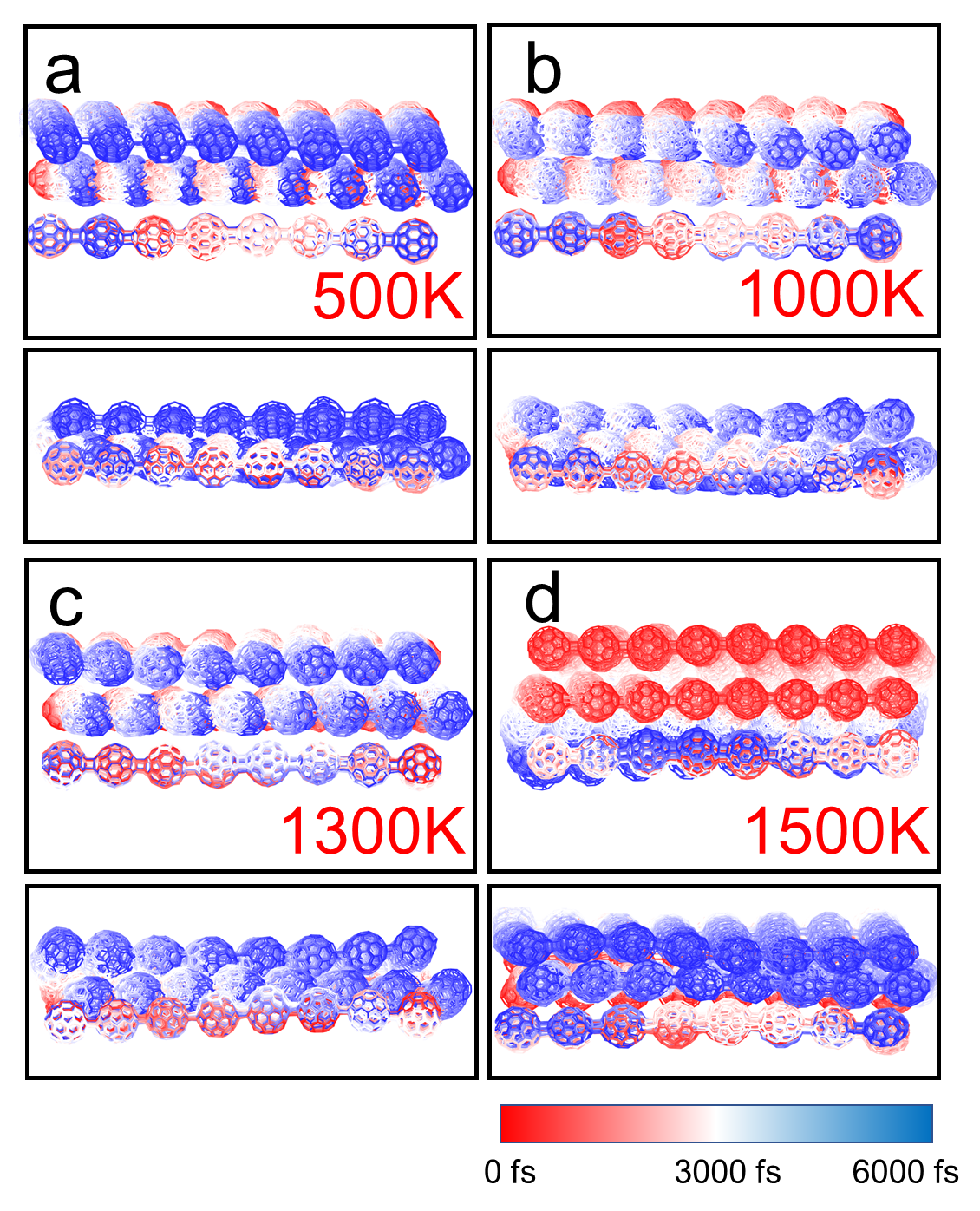


Fig. S9 AIMD trajectories of qTP-3 at 500, 1000, 1300 and 1500K, respectively. Red and blue represent structures in the early and late stages of the simulation, respectively.
